# Design and Fabrication of Flexible Naked-Eye 3D Display Film Element Based on Microstructure

**DOI:** 10.3390/mi10120864

**Published:** 2019-12-09

**Authors:** Axiu Cao, Li Xue, Yingfei Pang, Liwei Liu, Hui Pang, Lifang Shi, Qiling Deng

**Affiliations:** Institute of Optics and Electronics, Chinese Academy of Sciences, Chengdu 610209, China; longazure@163.com (A.C.); xueli2553@163.com (L.X.); yfpang7647@163.com (Y.P.); liuliweineko@163.com (L.L.); ph@ioe.ac.cn (H.P.); dengqiling@ioe.ac.cn (Q.D.)

**Keywords:** naked-eye 3D, microstructure, flexible, film, fabrication

## Abstract

The naked-eye three-dimensional (3D) display technology without wearing equipment is an inevitable future development trend. In this paper, the design and fabrication of a flexible naked-eye 3D display film element based on a microstructure have been proposed to achieve a high-resolution 3D display effect. The film element consists of two sets of key microstructures, namely, a microimage array (MIA) and microlens array (MLA). By establishing the basic structural model, the matching relationship between the two groups of microstructures has been studied. Based on 3D graphics software, a 3D object information acquisition model has been proposed to achieve a high-resolution MIA from different viewpoints, recording without crosstalk. In addition, lithography technology has been used to realize the fabrications of the MLA and MIA. Based on nanoimprint technology, a complete integration technology on a flexible film substrate has been formed. Finally, a flexible 3D display film element has been fabricated, which has a light weight and can be curled.

## 1. Introduction

With the development of science and technology, people are hoping to truly restore three-dimensional (3D) information of the object space. As a result, 3D display technology has emerged with the times and has become a research hotspot in the field of image displays [1,2,3,4,5,6].

As Wheatstone invented the first stereoscopic picture viewer in 1838, the technology of 3D displays has been developed for nearly 200 years [7]. In this development process, head-mounted 3D display technology is very mature in principle and technology [8,9,10,11], and there are a large number of commercial products. However, due to the need of wearing equipment, it is always inconvenient. Meanwhile, long-term use depending on the binocular parallax principle will lead to viewing fatigue, and viewers will feel dizzy. Therefore, it is an inevitable trend for the future development of naked-eye 3D display technology without wearing equipment.

Research groups have developed a variety of naked-eye 3D display technologies, including the grating 3D display technology of binocular parallax, holographic technology, and integrated imaging technology. Grating 3D display technology uses the principle of binocular parallax to produce a 3D sensation [12,13]. This technology has the advantages of low cost, simple structure, and easy implementation. However, because the left and right parallax images cannot be completely separated, the viewing area of the viewer is limited, and the 3D image can only be viewed in a relatively fixed position, lacking freedom. Therefore, it is only suitable for a single user and small range of motion.

By using the interference principle, holographic technology interferes the light wave reflected by the object with the reference light wave, and records it in the form of interference fringes to form a hologram [14]. When the hologram is illuminated by a coherent light source, the original light wave will be reproduced based on the diffraction principle, to form a realistic 3D image of the original object. However, the production of high-quality optical holograms requires a high-coherence laser, shockproof platform, and precise optical path setting. In addition, the ambient air flow will also affect the successful recording of holograms. Later, with the development of digital holography technology, using charge coupled devices (CCDs) instead of ordinary holographic recording materials to record holograms and using computer simulations instead of optical diffraction to realize object reproduction, the digitization of the whole process of hologram recording, storage, processing, and reproduction can be realized [15]. However, the resolution of digital holography using CCDs to record coherent light waves cannot be compared to that of holographic dry plates, so the resolution of holograms is relatively low, which seriously affects the image clarity.

The computer-generated hologram, which combines digital computing with modern optics, encoding the complex amplitude of the object light wave from a computer to computer-generated hologram (CGH), has unique advantages and good flexibility [16]. However, there is a large amount of data information and processing time for 3D objects, so it is necessary to select appropriate algorithms and coding methods to overcome [5,17]. In addition, the spatial light modulator plays a very important role in the experiment of CGH photoelectricity reproduction. It is necessary to overcome the influence of spatial light modulation on the quality of the reconstructed image [18,19]. At present, the CGH is still in the research stage of algorithm optimization to realize a 3D display of large scale and large field of view. It is still early to expect a commercial product based on holographic display devices.

Integrated imaging technology is also composed of two basic processes: Recording and reproduction. Unlike holographic technology, the recording and reproduction process do not require the participation of coherent light, which reduces the difficulty of the whole system. This technology was first proposed by Lippmann, a famous French physicist and Nobel Laureate in physics, in 1908 [20]. The microlens units in the microlens array (MLA) are used to record 3D object information from different perspectives to form a microimage array (MIA), and then the recorded MIA is placed on the focal plane of the MLA whose parameters are matched with a microlens in the recording process. The 3D image can be viewed by irradiating with scattered light according to the principle of optical reversibility and the fusion of the human brain. This technology can provide full parallax and a full-color image, without any special equipment, and the viewpoint provided is quasi-continuous. In a certain area, it can be viewed by many people, which has become an important development trend of naked-eye 3D displays.

The recording and reconstruction of 3D objects are formed through the interaction of tens of thousands or even hundreds of thousands of microimages. A camera array can be used to record the MIA. This method requires a large number of expensive and complex camera equipment, and the mechanical error between camera equipment will also affect the final imaging effect [21,22]. The optical recording method [23] uses an MLA to record the MIA, which is easy to be affected by surrounding environmental conditions such as brightness, sensitivity, and uniformity. The experimental operation is difficult, and the adjacent images are prone to crosstalk, resulting in a poor imaging effect of the final MIA. Then, the acquisition of the MIA based on 3D graphics software is proposed [24], which can realize the free crosstalk and high-resolution recording of the MIA. In addition, the display screen is generally used to display the recorded microimage array in 3D image reproduction. At present, the screen resolution of the mainstream high-definition display screen is 1920 × 1080, and the number of pixels per inch is 89, i.e., 89 ppi. Compared to the resolution limit of the human eye of 300 ppi, the resolution is still low. The granular distortion effect will be visible at a certain distance.

In this paper, based on the integrated imaging technology, a flexible naked-eye 3D display film element based on microstructures has been designed and fabricated, which can achieve the 3D imaging effect with a resolution higher than the human eye resolution limit of 300 ppi, and has the advantages of curling and light weight. The main arrangement of this paper is as follows: The second part describes the structure design and imaging principle of the 3D display film element. The third part presents the structural design of the MLA and acquisition of the MIA. The fourth part describes the preparation and integration of the microstructure, and the final part presents the summary of the whole paper.

## 2. Structural Design and Imaging Principle

**Figure 1:**
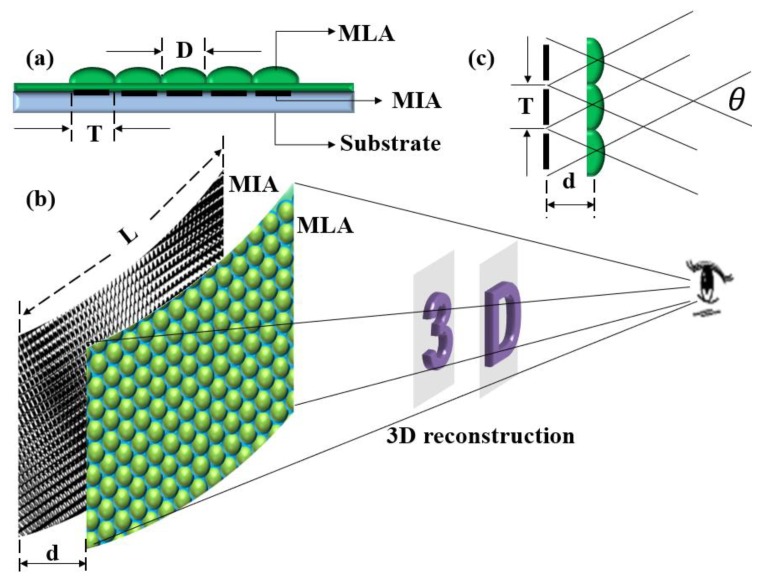
Flexible naked-eye 3D display film element: (**a**) structure composition; (**b**) imaging principle; (**c**) viewing angle.

The structure of the flexible naked-eye 3D display film element consists of three parts, namely, an MLA, MIA, and flexible substrate material, as shown in Figure 1a. The MIA is imaged by the MLA with different viewing angle information. The sub-images of each imaging channel are fused to form a 3D display effect. The human eye can watch the 3D image of the object in front of them, as shown in Figure 1b.

The aperture (*D*) of the microlens is the same as the size (*T*) of the microimage unit, and the distance (*d*) between the MLA and MIA is the effective focal length (*f*) of the microlens. A single microlens can image the corresponding microimage independently, and several sub-images are fused to form a 3D effect. According to the theory of Gauss optics, when the distance between the MLA and MIA is the focal length of the microlens, the image distance is infinite, which means that light from any angle on the MIA is refracted by the MLA and then emitted as parallel light. Therefore, the number of pixels of the reconstructed 3D image is determined by the number of MLAs (*n* × *n*). Finally, the imaging resolution (Re) of the element can be calculated according to Equation (1), where *L* is the size of the MIA. *L* can be obtained by multiplying the array number (*n* × *n*) of microimages by the size of the microimage unit (*T*). The viewing angle of the film element (Figure 1c) can be obtained from Equation (2). As the focus display mode [25] is used in this paper, the 3D depth range (Δ*Z*) is determined by Equation (3), where *P_I_* is the pixel size of the microimage.

(1) Re=nL,

(2) $$\theta =2\arctan(\frac{T}{2d}),$$

(3) $$\Delta Z=2\frac{dD}{P_{I}}.$$

## 3. Structural Design of Microlens Array (MLA) and Acquisition of Microimage Array (MIA)

### 3.1. Structural Design of MLA

Due to the MLA being the key imaging element, the reasonable design of the parameters of the MLA, such as the aperture, focal length, and array number, is related to the integration of the whole element and the quality of the 3D image. For a miniaturized 3D film element, when the microlens is designed with a large aperture or a small number of MIAs, the resolution of images from all perspectives of the 3D image will be very low, which makes the viewing effect worse. In order to meet the requirement of the human eye resolution of 300 ppi, the structural parameters of the microlens are designed as follows: (1) the material of the microlens is a photosensitive adhesive (NOA61), and the refractive index is 1.56 in the visible light band; (2) the aperture (*D*) of the microlens is 80 μm; (3) the curvature radius of the microlens is 47.5 μm; (4) the focal length (*f*) of the microlens is 85 μm; (5) the sag height of the microlens is 22 μm; (6) the array number of the microlens is 250 × 250. Then, the imaging resolution is calculated as 317 ppi, which is higher than the human-eye resolution limit. The viewing angle (*θ*) is about 50°. At this time, the focal length of the microlens is very short, so the distance between the MLA and MIA is 85 μm. Thus, the whole element will reach the thin-film level.

### 3.2. Acquisition of MIA

The acquisition of tens of thousands or even hundreds of thousands of microimages has been carried out based on 3D graphics software. This method does not need complicated and expensive optical equipment, and can also avoid human and mechanical errors in the operation of optical equipment. With the use of computer memory, computer generation technology can generate microimages quickly, accurately, and in large quantities.

**Figure 2:**
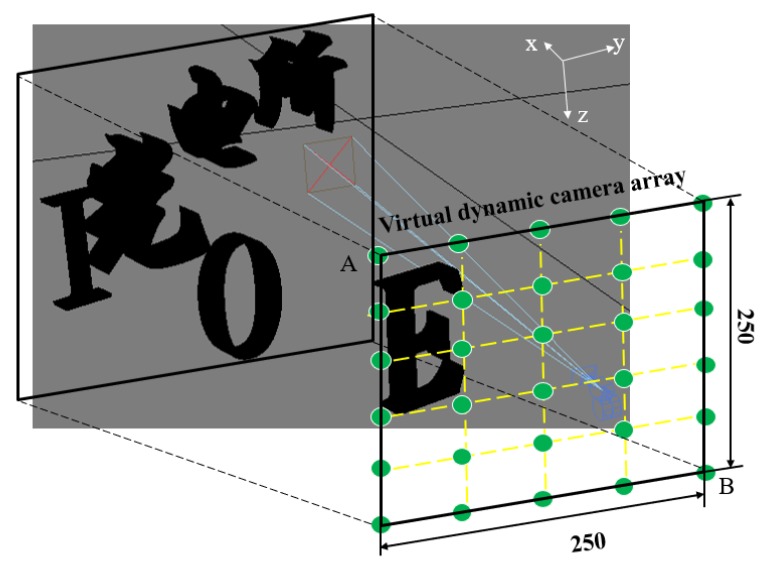
250 × 250 microimages acquired by dynamic camera.

Based on 3ds MAX software (3ds MAX 2009, San Rafael, CA, USA), a 3D scene was established, as shown in Figure 2. The scene contained the Chinese characters “光电所” and letters “IOE.” The central 3D coordinates of “光”, “电”, and “所” were (15.59 mm, −6.8 mm, 9.5 mm), (30.79 mm, 0, 9.5 mm), and (37.88 mm, 6.8 mm, 9.5 mm), respectively. The central 3D coordinates of “I”, “O”, and “E” were (37.35 mm, −6 mm, 13 mm), (23.43 mm, 0, 13 mm), and (12.48 mm, 6 mm, 13 mm), respectively. The virtual dynamic camera array was established to simulate the image acquisition process of the whole MLA, and the acquisition of the microimage was carried out for different 3D information from far to near. In the image acquisition, the 3D coordinates of the start point of the camera were A (0, −12.5 mm, 0), and the 3D coordinates of the end point were B (0, 7.42 mm, 19.92 mm). The field of view of the camera was 5° and the moving interval was 80 μm, which was matched with the structural parameters of the MLA. Finally, 250 × 250 microimages were acquired. The pixel number of the microimage was 40 × 40, and the pixel size of the microimage (*P_I_*) was 2 μm. Therefore, the 3D depth range could be calculated as 6.8 mm.

**Figure 3:**
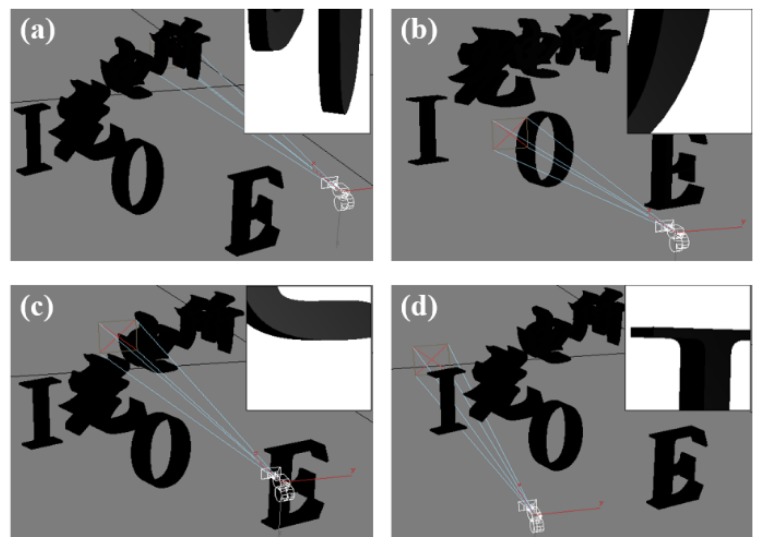
Imaging from different perspectives: (a) imaging from one prespective of “所”, (b) imaging from one prespective of “O”, (c) imaging from one prespective of “电”, and (d) imaging from one prespective of “I”.

In the process of MIA acquisition, the camera captured images of the 3D scene from different perspectives, as shown in Figure 3, to record the information at different perspectives. In addition, the obtained microimages are shown in the box in the upper right corner of the corresponding perspective. It can be seen that the microimages captured by the virtual camera have a very high image resolution and perfect image quality.

**Figure 4:**
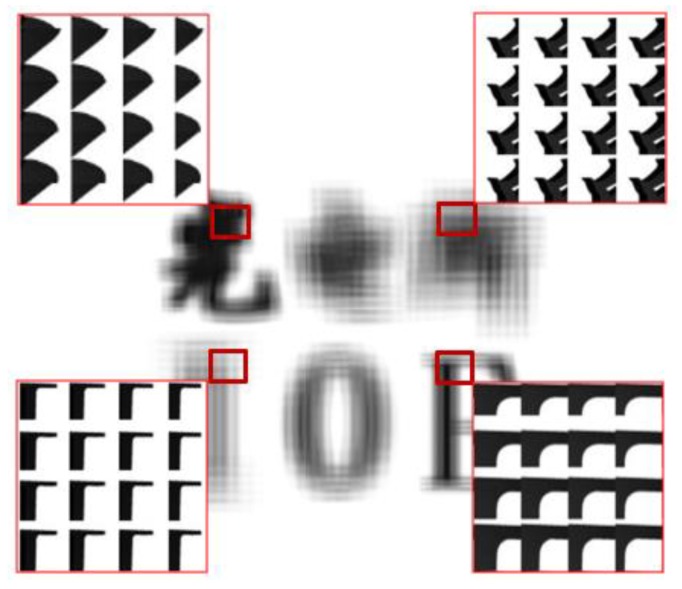
Microimage array (MIA) with different enlarged images of different regions.

Furthermore, 250 × 250 microimages captured by the camera were encoded and fused according to the arrangement of the MLA. The MIA was generated by computer processing, as shown in Figure 4. From the enlarged images of different regions, we can see that each microimage is different with information of different perspectives from the 3D scene.

## 4. Preparation and Integration

**Figure 5:**
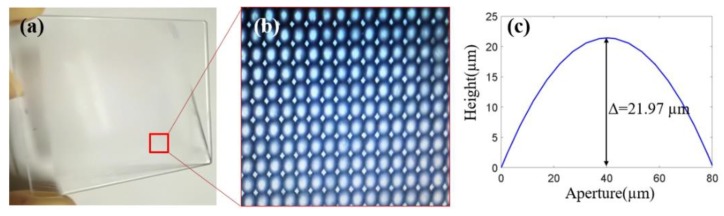
Preparation results of microlens: (**a**) prototype of microlens array (MLA); (**b**) micromagnifier of microlens; (**c**) surface profile of microlens.

**Figure 6:**
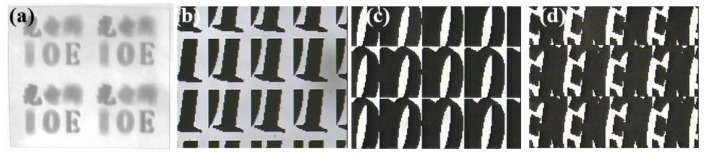
Preparation results of (**a**) MIA in different areas: (**b**) few microimages of “I”, (**c**) few microimages of “O”, and (**d**) few microimages of “E”.

There are two key microstructures in the 3D display element, which are the MLA and MIA. The MLA was prepared by photolithography and the hot melting method, and the preparation results are shown in Figure 5, which shows the prototype of the MLA (Figure 5a), micromagnifier of the microlens (Figure 5b), and surface profile of the microlens (Figure 5c). The sag height of the microlens was 21.97 μm, which is consistent with the design result. The MIA was also prepared by photolithography. The pattern was prepared on the substrate material by a series of processes such as exposure, development, and etching. The MIA and enlarged images of different regions of the MIA are shown in Figure 6. The substrate material was a polyethylene terephthalate (PET) film, which has characteristics of high toughness, smooth surface, and good light transmittance. The pattern was made by lithography with a high resolution of minimum linewidth of 2 μm, which is equal to the pixel size of the microimage.

**Figure 7:**
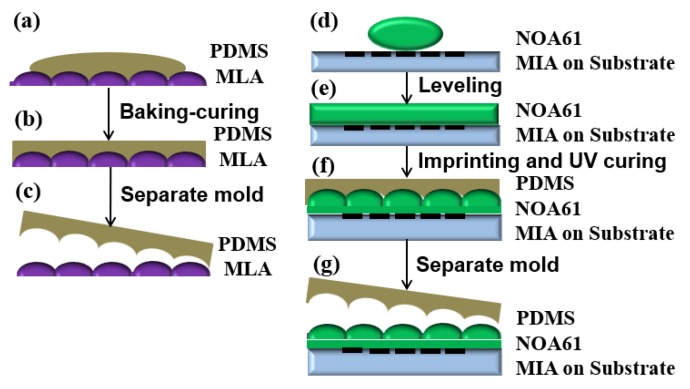
Integration of thin film element: (**a**) pouring of polydimethylsiloxane (PDMS) materials; (**b**) baking and curing; (**c**) generation of imprinting mold; (**d**) photosensitive adhesive (NOA61) dropped on the prepared MIA; (**e**) leveling; (**f**) imprinting and UV curing; (**g**) mold peeled off.

Subsequently, integration of the two key microstructures needs to be carried out. During the integration, the MLA and MIA need to be aligned one by one to realize 3D image reconstruction. In the experiment, nano-imprinting alignment technology was used to achieve alignment integration of the two microstructures, as shown in Figure 7.

First, the imprinting mold with the structural information of the MLA should be prepared. The imprinting mold is composed of polydimethylsiloxane (PDMS). The free energy of the interface of the PDMS mold is low and has chemical inertness. Therefore, the mold is easy to be separated when the mold is used to carry out the integration. During the mold preparation, PDMS stroma and curing agent were poured into a clean beaker at a volume ratio of 10:1, continuously stirring with a glass rod. A large number of bubbles were generated in the PDMS prepolymer until the bubbles disappeared gradually. In addition, the PDMS prepolymer was poured on the prepared MLA, as shown in Figure 7a. Then, the substrate was placed on the coater at a speed of 250 rpm with a time of 20 s, shaking off the excess PDMS. Subsequently, it was placed in a vacuum oven until all the bubbles disappeared for curing, as shown in Figure 7b. The baking temperature and time were set as 65 °C and 4 h, respectively. Finally, the PDMS imprinting mold with negative structural information of the MLA on the surface could be peeled off from the MLA, as shown in Figure 7c.

**Figure 8:**
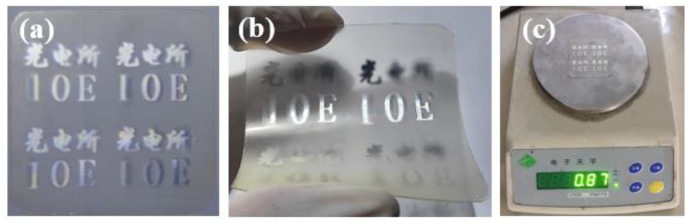
Integration of thin film element: (**a**) planar display; (**b**) curved display; (**c**) weight.

Secondly, the structural information of the MLA should be imprinted on the surface of the MIA by using the imprinting mold to realize the integration of the MLA and MIA. The process flow was as follows: 

First, the photosensitive adhesive (NOA61) was dropped on the prepared MIA, as shown in Figure 7d. After it was leveled (Figure 7e), the imprinting mold was used to imprint photosensitive adhesive. During imprinting, the high-precision alignment device was used to align the microlenses with the microimages one by one. On the basis of alignment, the photosensitive adhesive was irradiated by ultraviolet light with a wavelength of 365 nm until it was cured, as shown in Figure 7f. Finally, the PDMS mold was peeled off (Figure 7g) to obtain an integrated 3D display film element, as shown in Figure 8. 

**Figure 9:**
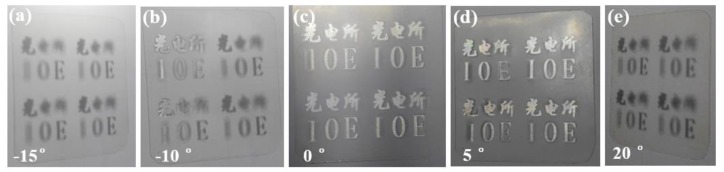
3D effects from different viewing angles: (**a**) −15°; (**b**) −10°; (**c**) 0°; (**d**) 5°; (**e**) 20°.

Figure 8a shows the 3D effect of the planar display, and the 3D display effect can be seen from various angles. Figure 8b shows the 3D display effect after bending of the element. Meanwhile, the weight of the 3D display element on the flexible substrate has been characterized, which is less than 1 g, as shown in Figure 8c, reaching the lightweight level. Figure 9 shows the 3D effects from different viewing angles.

## 5. Conclusions

In this paper, we propose to design and fabricate a 3D display film element based on microfabrication. The imaging resolution is higher than that of the human eye at 300 ppi. At the same time, the element has the characteristics of miniaturization and light weight, which can be applied to product packaging, handicrafts, anti-counterfeiting, and other industries. Using the 3D display effect to replace the original two-dimensional image display has a certain market application prospect, and also lays the technical foundation for wearable display equipment.
